# Clinical Outcomes of the U-Linear Split Thickness Skin Graft Technique for Reconstruction for Ear Canal Stenosis

**DOI:** 10.22038/ijorl.2025.87421.3932

**Published:** 2025

**Authors:** Kanokkarn Mahawerawat, Pornthep Kasemsiri, Chonthicha Chit-uea-ophat

**Affiliations:** 1 *Department of Otorhinolaryngology, Khon Kaen Hospital,* *Khon Kaen, Thailand.*; 2 *Department of Otorhinolaryngology, Buengkan Hospital, Buengkan, Thailand.*; 3 *Department of Otorhinolaryngology, Srinagarind Hospital, Faculty of Medicine, Khon Kaen University, Khon Kaen, Thailand.*; 4 *Department of Otorhinolaryngology, Chaiyaphum Hospital, Chaiyaphum, Thailand.*

**Keywords:** Audiometry, Canaloplasty, External auditory canal stenosis, Split thickness skin graft

## Abstract

**Introduction:**

Canaloplasty is challenging because of the high rate of postoperative restenosis. The aim of this study was to evaluate the outcomes of using a U-Linear split-thickness skin graft (U-Linear STSG), a novel modified graft placement technique for canaloplasty.

**Materials and Methods:**

A retrospective cross-sectional study was conducted on patients who underwent canaloplasty between January 2013 and December 2023. The medical records of external auditory canal stenosis patients who underwent canaloplasty were reviewed. The data collected included patient demographics, surgical approaches, postoperative outcomes, and audiometric findings. The outcomes of reconstruction for ear canal stenosis using a U-Linear STSG were compared with those using a reconstructed local flap. Statistical analyses included chi-square tests for categorical data and independent t tests for continuous data.

**Results:**

Thirty-six patients with external auditory canal stenosis underwent reconstruction; 17 patients underwent reconstruction with a U-Linear STSG, and 19 patients underwent reconstruction with a local flap. A review of the clinical outcomes revealed that postoperative restenosis was significantly less common in the U-Linear STSG group than in the local flap group (p < 0.05). In terms of audiometry, the postoperative air‒bone gap in the U-STSG group was slightly greater than that in the local group. No serious complications were observed in either group.

**Conclusion:**

A U-Linear STSG can be simply and feasibly applied in reconstruction for ear canal stenosis, with no major complications.

## Introduction

External auditory canal stenosis is a rare condition whose aetiology can be classified as either congenital or acquired. Acquired external auditory canal stenosis can result from various factors that trigger inflammatory tissue responses, ultimately leading to narrowing of the external auditory canal. 

The incidence of acquired external auditory canal stenosis is approximately 0.6 per 100,000 individuals (1). 

The causes of acquired external auditory canal stenosis include chronic otitis externa, dermatologic diseases, iatrogenesis (resulting from previous ear surgery or radiotherapy), trauma, and tumours (2). 

The consequences of external auditory canal stenosis include hearing loss and the possible development of a cholesteatoma. 

Lavy and Fagan (3) reported conductive hearing loss in patients with an air‒bone gap of approximately 30–40 dB, along with a flat tympanogram and the absence of stapedial reflexes. Additionally, progressive hearing loss of 15 dB to 40 dB was observed over an 8-year period (4).

 Becker and Tos (1) reported that, in patients who develop a cholesteatoma, 9% of cholesteatomas develop behind the site of stenosis.With respect to treatment, surgery is the primary modality of choice (1,3,5-7). Various surgical techniques have been introduced for canaloplasty. Bajin et al. (8) reported that both endaural and postauricular approaches have been utilized during surgery. Complete resection of the fibrous plug at the stenosis site is recommended (1,4,9-11). 

The degree of resection of the stenosed segment depends on the extent of the disease to prevent postoperative restenosis; therefore, other procedures, such as meatoplasty, tympanoplasty, and the creation of a bony canal by drilling, may be necessary in conjunction with canaloplasty. After the fibrous plug is removed, a flap or graft is used to cover the bare ear canal to reduce the probability of restenosis. However, the best flap or graft to use is still unclear. 

Numerous types of grafts have been discussed, but the optimal placement remains unclear. Thus, a simple canaloplasty technique using a U-shaped split-thickness skin graft is presented in this study. This technique is not difficult even for novice surgeons. 

## Materials and Methods

The retrospective study was conducted on patients who underwent canaloplasty between January 1, 2013, and December 31, 2023. The medical records of patients who underwent canaloplasty were reviewed. During the early period of the study, canaloplasty was performed using a local flap, and the U-Linear split-thickness skin graft was modified and developed during the later period.

*Surgical techniques*

After induction of general anaesthesia in the patient, microscopic canaloplasty was performed via the postauricular or endaural approach, depending on the extent of the disease.

*Using a local flap*

The superficial epithelial lining of the fibrous tissue and skin flap of the ear canal was raised, after which the fibrous plug in the ear canal was excised. Following the complete removal of the fibrous plug, the epithelial lining was replaced, and the skin of the canal was rotated to cover the bare area of the canal. A stent was placed 4–6 weeks after surgery to prevent restenosis.

*Using a U-Linear split-thickness skin graft (U-Linear STSG)*

**Fig 1:**
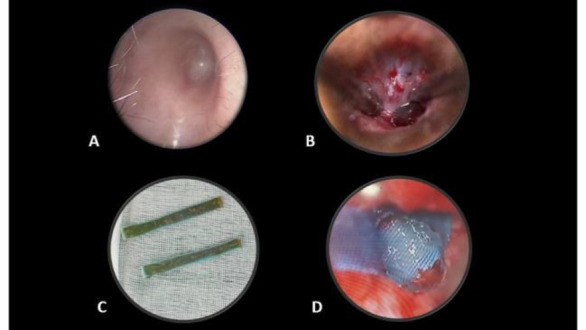
After examination of external auditory canal stenosis (A), an incision was made 1 mm lateral to the fibrous tissue, followed by complete resection of the fibrous plug (B). The split-thickness skin graft (STSG) was harvested and placed with the dermal side facing up on sterile rayon. The STSG was sliced into three or four sections, each section having a width of 3 mm and a length of 60 mm (C). The STSG was placed in an overlapping “U” shape in the ear canal using rayon, ensuring contact with the bare area (D). Several small cotton balls soaked in antibiotics were packed on the rayon in the ear canal to stabilize the grafts.

**Fig 2:**
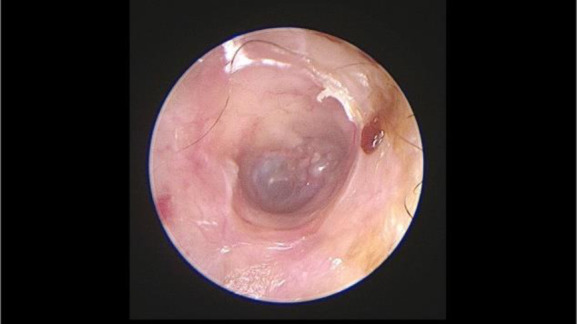
Six weeks after U-Linear STSG placement, an examination was performed to assess the surgical area following the removal of the cotton packing.

An incision was made 1 mm lateral to the fibrous tissue, followed by complete resection of the fibrous plug and drilling of the bony external auditory canal. A split-thickness skin graft (STSG) was harvested and placed with the dermal side facing upwards on sterile rayon. The rayon was custom-prepared from linen cloth that had been sterilized rather than commercially manufactured. The STSG was divided into three or four pieces, each measuring 3 mm in width and 60 mm in length. The STSG was placed with the rayon covering the bare canal. The rayon facilitated placement of the graft into the ear canal in a “U”-shape, ensuring that the graft remained in contact with the exposed area. The epithelial surface of the STSG was oriented towards the rayon, whereas the subcutaneous surface was positioned in direct contact with the bony wall of the ear canal. Each piece of the STSG was placed according to the contour of the canal (Figure 1). Several small cotton balls soaked in antibiotics were packed over the rayon in the ear canal to stabilize the grafts. Three to six weeks after surgery, the cotton balls and rayon were gently removed. All the patients who underwent canaloplasty received oral antibiotics for one to two weeks and antibiotic ear drops for three to six weeks. After the removal of the stent or the cotton packing, an endoscope examination was conducted to assess the area (Figure 2), and hearing was evaluated through audiometry.

*Analyses*

Descriptive data are presented as percentages and means ± SDs. Additionally, inferential statistics (chi-square test and independent t test) were used to compare the data between the local flap group and the U-Linear STSG group. A p value < 0.05 indicated statistical significance. The data were analysed with STATA (v 16.1: Stata Corp. 2019, Texas, USA).

*Ethical review*

This retrospective study was reviewed and approved by the Human Ethics Research Committee of Khon Kaen Hospital (KEXP 67016) and was conducted in accordance with the Declaration of Helsinki. Owing to the retrospective nature of the study, the Human Ethics Research Committee of Khon Kaen Hospital waived the requirement for informed consent.

## Results

Thirty-six medical records of acquired external canal stenosis patients (18 men and 18 women) were reviewed. The demographic characteristics of both groups are presented in Table 1. 

There was no significant difference in the demographic data between patients treated with a local flap and those treated with a U-shaped linear STSG. The most common cause of external canal stenosis was infection, which was found in 70.6% of the patients in the U-Linear-STSG group and 94.7% of those in the local flap group.

**Table 1:** Demographic data

**Characteristics**	**U-Linear STSG (N = 17)**		**Local flap (N = 19)**	
	**N**	**%**	**N**	**%**
Sex				
Male	9	52.9	9	47.4
Female	8	47.1	10	52.6
Age (years; mean)	43.1±18.6		43.4±17.2	
Underlying disease				
Hypertension	3	17.7	0	0
Dyslipidaemia	3	17.7	0	0
Chronic kidney disease	2	11.8	0	0
Ischaemic heart disease	1	5.9	1	5.3
Cause of stenosis				
Infection	12	70.6	18	94.7
Trauma	2	11.8	0	0
Malignancy	1	5.9	0	0
Surgery	1	5.9	1	5.3
Fibrous dysplasia	1	5.9	0	0
Duration of stenosis (months; mean)	15.4±17.0		18.6±19.6	

The surgical data were analysed (Table 2) and found to be similar between the two groups; however, there were two cases of middle ear cholesteatoma and one case of middle ear granulation that required mastoidectomy in the U-Linear STSG group. 

These factors led to longer operative times in the U-Linear STSG group than in the local flap group (P < 0.05). The final surgical outcomes included 5 cases of restenosis in the U-Linear STSG group and 13 cases in the local flap group (P< 0.05).

**Table 2:** Surgical data

**Characteristics**	**U-Linear STSG (N = 17)**		**Local flap (N = 19)**		**P - value**
	**N**	**%**	**N**	**%**	
Surgical time (min; mean)	108.3±28.2		48.9±23.4		< 0.001
Intraoperative comorbidity findings						
Tympanic membrane perforation	3	17.7	2	10.5	0.087
Canal cholesteatoma	4	23.5	5	26.3	
Tympanic membrane perforation with cholesteatoma	2	11.8	0	0	
Granulation	3	17.7	0	0	
Conjunctional surgical procedure						
Tympanoplasty	6	35.3	2	10.5	0.045
Mastoidectomy	2	11.8	0	0	
Tympanomastoidectomy	1	5.9	0	0	
Explore middle ear	1	5.9	0	0	
Meatoplasty	0	0	1	5.3	
Postoperative restenosis	5	33.3	13	72.2	0.038
Follow up period (months; mean)	14.3±15.8		18.9±19.8		0.450

In terms of audiometry, as shown in Table 3, the preoperative air‒bone (AB) gap in both groups was nearly the same. After surgery, the AB gap in the U-Linear STSG group was slightly greater than that in the local flap group (P=0.762). No major complications were observed during the follow-up period.

**Table 3:** Audiometry data

**Characteristics**	**U-Linear STSG (N = 17)**		**Local flap (N = 19)**		**P - value**
	**Mean (dB)**	**SD**	**Mean (dB)**	**SD**	
Preoperative AC	57.2	±19.2	58.1	±24.6	0.925
Preoperative BC	24.1	±14.7	26.1	±18.5	0.773
Preoperative AB gap	33.2	±11.6	32.0	±13.0	0.825
Postoperative AC	43.9	±24.8	51.9	±25.3	0.503
Postoperative BC	22.7	±16.3	28.3	±19.4	0.509
Postoperative AB gap	21.2	±16.6	23.6	±17.7	0.762

## Discussion

Canaloplasty for external auditory canal stenosis remains challenging, particularly in regard to preventing restenosis. In previous studies, researchers proposed various techniques, including allowing for bare bone healing (12,13), using stents (14,15), applying skin grafts, and utilizing local flaps. Secondary bare bone healing is associated with a high risk of scar contracture, which can contribute to restenosis. In terms of grafts and flaps, the present study reveals that the restenosis rate associated with placement of a U-Linear STSG is significantly lower than that associated with placement of a local flap. A review of the literature revealed that graft placement appears to have a high success rate. Scaria et al. (16) reported the endoscopic removal of obstructive tissue and re-epithelialization using an STSG in 8 ears, with no restenosis observed in any of the patients; however, they did not mention the placement of an STSG. Schwarz et al. (17) studied 16 patients with external auditory canal stenosis who underwent canaloplasty with an STSG and reported a high success rate, with a reatresia rate of 10.5% and a restenosis rate of 18.8%. They described placing an STSG on the eardrum and on any de-epithelialized areas of the external auditory canal; however, placement of the STSG on deepithelialized areas of the external auditory canal was challenging for novice surgeons. Therefore, we modified graft placement by placing the STSG in a U shape, which is a simpler method. A restenosis rate of 33.3% was observed, comparable to that reported in a previous study. Our placement of the STSG appears similar to the overlapping technique described by Zou et al. (18), in which the STSG was overlapped to create scar anchor points and reduce the risk of skin contraction, helping to prevent restenosis and therefore increasing the success rate of grafting. Nonetheless, we did not implant an artificial drum ring and provided effective support with a postoperative model stent for the external auditory canal; thus, our success rate was lower than that reported in Zou's study (18). 

There are two limitations in the present study. First, only a few external auditory canal stenosis patients were included, and some data could not be retrieved because of the retrospective nature of the study. Second, the follow-up period varied, resulting in data inconsistencies. Future well-designed studies are necessary to improve the robustness of the findings and to provide more comprehensive insights into the outcomes of treatment of patients with external auditory canal stenosis.

## Conclusion

The placement of a U-Linear STSG is straightforward and feasible for the repair of external auditory canal stenosis. This technique provides a favourable safety profile, with no incidence of major complications. A U-Linear STSG is a compelling option for otologists addressing external auditory canal stenosis.

## Abbreviations:

STSG: split-thickness skin graft AB gap: air–bone gap
